# Adaptive Identification of Food Sweetness Concentration: An Electroencephalogram Feature Classification Network Under Taste Stimulation

**DOI:** 10.3390/foods14223855

**Published:** 2025-11-11

**Authors:** He Wang, Hong Men, Yan Shi

**Affiliations:** 1 School of Automation Engineering, Northeast Electric Power University, Jilin 132012, China; 1202300098@neepu.edu.cn (H.W.); shiyan@neepu.edu.cn (Y.S.); 2 School of Transportation, Jilin Tiedao University, Jilin 132299, China; 3 Advanced Sensor Research Institution, Northeast Electric Power University, Jilin 132012, China

**Keywords:** food sweetness concentration, EEG detection, EEG feature calculation module, deep learning

## Abstract

Detecting and identifying consumers’ perception of food sweetness can help guide the optimization of food formulations. Electroencephalogram (EEG) detection can capture changes in brain electrical activity in response to different sweet taste stimuli. In this work, we employ EEG detection and propose an EEG Feature Calculation and Classification Network (EFCC-Net) to recognize taste EEG signals under different sweetness concentration stimuli. First, taste-related EEG data from a subject group under varying sweetness concentration stimuli are collected. Then, an EEG Feature Calculation Module (EFCM) is proposed, which utilizes convolutional kernels of different sizes to compute local features from both temporal and spatial dimensions of EEG data. A lightweight self-attention mechanism is employed to compute global features, and a multi-branch computation approach is adopted to enhance feature extraction capability. Next, based on EEG topographic maps, qualitative analysis is conducted to examine differences in brain region activation under varying taste concentrations. Finally, leveraging the proposed EFCM, the EFCC-Net is designed to classify EEG data corresponding to different sweetness levels. Through structural optimization, ablation experiments, and comparisons with state-of-the-art EEG classification methods, EFCC-Net achieves the best classification performance, with an accuracy of 96.57%, a precision of 96.58%, and a recall of 96.53%, while also demonstrating superior stability.

## 1. Introduction

Food sweetness is one of the most important taste perceptions in human food experiences, playing a crucial role in food development and improvement [1,2]. Detecting the sweetness intensity of different foods ensures taste safety and adaptability, while a deeper understanding of the gradient recognition of sweetness intensity can help optimize food formulations and guide product development [3,4]. Manufacturers can adjust formulations based on consumers’ taste sensitivity, thereby enhancing flavor experiences [4].

Traditionally, the assessment of food sweetness has primarily relied on sensory evaluation techniques, such as difference testing and descriptive analysis conducted by trained panelists. While these methods provide direct consumer insights, they are inherently subjective and susceptible to individual biases, psychological states, and cultural backgrounds. Moreover, they cannot capture the underlying neurophysiological processes of taste perception. As a semi-objective physiological monitoring technique, Electroencephalography (EEG) technology provides objective, quantifiable neurophysiological data that overcomes the subjectivity and personal biases inherent in sensory evaluation. In emotion recognition, EEG signals are used to decode human emotional states [5]. In brain–computer interfaces, it enables control of external devices through motor imagery tasks [6]. In cognitive neuroscience, it investigates the mechanisms of attention, memory, and decision-making [7]. In clinical diagnosis, it aids in the detection and monitoring of neurological disorders such as epilepsy and sleep disorders [8]. The successful application of EEG across these diverse domains supports its feasibility and potential for concentration perception in response to taste stimuli.

EEG can detect changes in brain activity in response to different taste stimuli, thereby overcoming the limitations of traditional subjective methods and enabling an objective assessment of taste responses [7,9]. By studying the brain’s EEG signals to different concentrations of sweetness, we can gain a better understanding of consumers’ neural reactions and preferences [1,10,11]. This helps food researchers design products that are both healthy and capable of activating the brain’s reward mechanisms, reducing sugar intake while improving dietary structures and overall health. EEG detection technology captures changes in brain electrical activity in response to different taste stimuli, providing a semi-objective and physiological detection method [12]. By comparing EEG signals under varying sweetness concentrations, researchers can gain deeper insights into the neural dynamics and spatiotemporal characteristics of taste perception, offering objective evidence for the neural mechanisms of taste information transmission and integration. This provides strong data support for optimizing product flavors in food science, ensuring food safety, and scientifically formulating nutritional balance.

During the process of EEG detection, EEG signals are characterized by high dimensionality, multi-channel properties, and time-varying dynamics, making it a significant challenge to extract discriminative features from such complex data [13,14]. Traditional EEG information processing methods primarily involve two stages: feature computation and pattern recognition [15]. Jenke R et al. analyzed various EEG signal feature extraction methods, such as Fourier transform and discrete wavelet transform, concluding that multivariate feature selection performs better in emotion recognition tasks [16]. Hou H et al. proposed a method for EEG signal processing based on the double square electrode sequence, which provided a solution for the classification of highly similar EEG signals by constructing a double square feature set to characterize the electrode sequence features [17]. Mhaskar C et al. combined K-means clustering and principal component analysis to compare the degree of EEG relaxation induced by different aromatic stimuli [18]. Cui Z et al. proposed an umami evaluation model based on EEG and machine learning. Four optimal classification algorithms (μ support vector machine, K-nearest neighbor, bag method and random classification) were selected as sub-models, and the confidence probabilities of the four sub-models were fitted by support vector machine to obtain the optimal classification method [19]. Clearly, traditional EEG signal recognition methods involve complex processes, requiring continuous experimentation to determine optimal feature extraction and classification techniques, with low adaptability, leading to poor recognition stability.

With the advancement of deep learning technologies, researchers have begun exploring end-to-end processing methods [11,20,21]. Huang Q et al. proposed a multi-level attention-based multi-scale fusion temporal convolutional network for decoding MI-EEG signals [14]. Xing M et al. used convolutional neural network (CNN) and bidirectional long term memory hybrid model to realize the emotion recognition of olfactory enhanced EEG signals [22]. Ma et al. proposed a Spatial-frequency Shifted Windows Time Self-attention Neural Network, which efficiently leverages valuable time, spatial, and frequency domain information present in EEG signals [23]. Tong C et al. employed a self-attention mechanism to capture the temporal dynamics of EEG signals, significantly improving classification accuracy compared to traditional power spectral density and Hjorth parameter methods [24]. Gao H et al. introduced a multi-scale CNN-based EEG classification method incorporating residual connections to identify basic human taste perceptions [9]. Li D et al. proposed a spatial-frequency convolutional self-attention network that addresses the challenge of multi-band information integration and notably enhances emotion recognition performance under taste stimulation [25]. Martins et al. employed Inception networks, data augmentation, and transfer learning for the task of EEG-based diagnosis of photosensitivity [26]. Xia X et al. combined a frequency-band partitioned Transformer architecture, offering a novel technical pathway for EEG data mining and food sensory evaluation [27]. Additionally, they designed a local-global fusion Transformer network to strengthen the model’s capability in recognizing complex EEG responses [28]. Furthermore, they integrated channel attention and spatial attention mechanisms into a CNN structure for EEG channel selection, achieving improved classification performance [29]. Deep learning methods can establish nonlinear mappings from raw data to decision outputs through well-designed network architectures, offering strong adaptability and simplified data processing [30,31]. However, EEG data inherently possess distinct temporal and spatial structural characteristics, and current approaches still lack sufficient analysis of the relationship between local evoked potentials and global EEG information. In other words, existing analytical methods often fail to fully account for the unique properties of EEG data when constructing network architectures. Moreover, high-dimensional data not only increase computational complexity but also easily lead to overfitting issues. Therefore, developing effective analytical methods remains crucial for achieving accurate EEG signal decoding and recognition.

To address the challenges in objectively identifying sweetness concentrations and decoding the corresponding neural signatures, this study aims to develop an efficient EEG-based classification framework. The primary objectives and corresponding contributions of this work are threefold:

1. To visually elucidate the neural dynamics of sweet taste perception, we employ brain source localization to map the intensity, spatial distribution, and temporal dynamics of EEG under varying sweetness concentrations. This aims to verify the feasibility of qualitatively identifying different sweetness levels using EEG data.
2. To effectively extract discriminative features from the complex taste-EEG data, we propose a novel EEG Feature Computation Module (EFCM). This module is designed to capture multi-scale features through a multi-branch structure, convolutional layers for local features, a self-attention mechanism for global dependencies, and residual connections for stable training.
3. To achieve accurate and stable classification, we construct a lightweight EEG Feature Classification Network (EFCC-Net) based on the EFCM. The network’s performance and superiority are rigorously validated through structural optimization, ablation studies, and comparisons with state-of-the-art methods.

The rest of this paper is structured as follows: Section 2 introduces the materials and methods, including experimental instruments, procedures, and data processing methods. Section 3 presents the results and discussion, providing a qualitative analysis of EEG perception under different sweetness levels, structural optimization of EFCC-Net, ablation experiments, and performance comparisons.

## 2. Materials and Methods

### 2.1. Instrument and Sample

**Figure 1:**
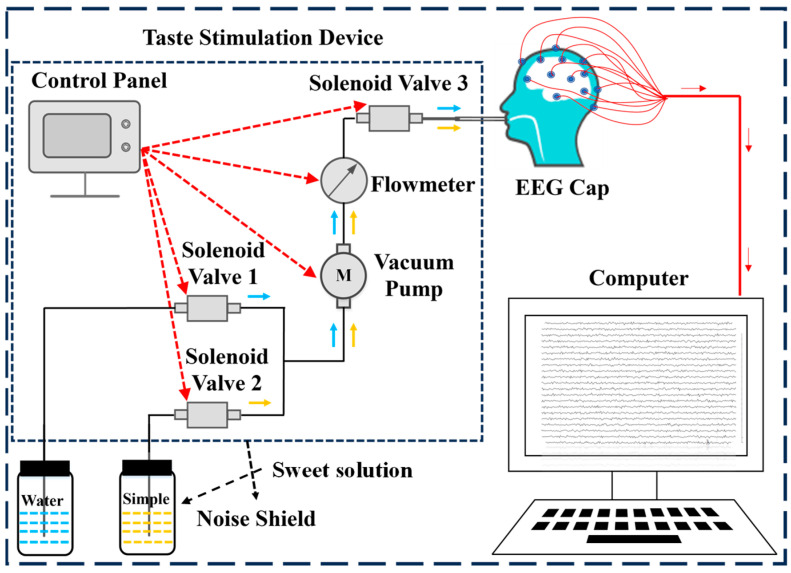
Structure of the EEG system for sweetness detection.

The EEG signals are acquired using an NCEP-P EEG and EP instrument (NCC Electronics Co., Ltd., Shanghai, China) at a sampling frequency of 256 Hz. According to the international 10–20 system, 21 channels (Fz, Cz, Pz, T3, T4, C3, C4, Fp1, Fp2, F7, F8, T5, T6, O1, O2, F3, F4, P3, P4, A1, A2) are positioned on an EEG cap (Greentek Pty. Ltd., Wuhan, China). We employ a self-developed high-precision, low-noise, and highly stable taste stimulation device designed to collect EEG signals from subjects under sweet taste stimulation. The structure of the equipment is shown in Figure 1, mainly composed of STM32F103ZE control panel (Dongguan Wildfire Technology Co., Ltd., Dongguan, China), S15S-53J micro vacuum pump (Chengdu Hailin Technology Co., Ltd., Chengdu, China), SFO-1037V-01 solenoid valve (Dongguan Sfan Electronic Technology Co., Ltd., Dongguan, China) and AB32-S21P020C-11R liquid flowmeter (ODE Limited, Hong Kong, China). The control board enables precise regulation of liquid flow rate and volume, solenoid valve switching, and vacuum pump operation. Additionally, noise is effectively minimized through soundproof enclosures and acoustic foam.

In this study, sucrose (BIOSHA, purity ≥ 99.9%) is selected as the sweet stimulus to analyze EEG activity under sweet taste stimulation. Two hours prior to the experiment, five different concentrations of sweet solutions are prepared. 0.3 g, 0.6 g, 1.2 g, 2.4 g, and 4.8 g of food-grade sucrose are accurately weighed and completely dissolved in 100 g of distilled water, labeled as concentrations 1 to 5, respectively. Pure distilled water (labeled as concentration 0) served as the control. All prepared solutions are stored in a 37 °C constant-temperature incubator to maintain stable temperature conditions approximating human oral temperature.

### 2.2. Taste-Evoked EEG Experiment

Prior to the experiment, we screened 20 right-handed, non-smoking participants (10 males and 10 females, aged 22–25 years). The selection of this age range was to minimize age-related variations in taste sensitivity and ensure a homogeneous subject pool. Upon confirming the feasibility of the approach, our subsequent work will involve recruiting participants with diverse demographic profiles and age ranges to validate the effectiveness of the proposed method. All participants were confirmed to be healthy with no reported history of taste, olfactory, or neurological disorders. To ensure data quality, all participants received standardized training before the experiment. This included a detailed explanation of the procedure and a demonstration of the required behavior during EEG recording, such as remaining still, keeping their eyes closed, and refraining from swallowing. A practice trial using distilled water and sweet solution were conducted to familiarize participants with the process.

The laboratory temperature was strictly maintained at 21 ± 2 °C to ensure experimental stability. During the testing procedure, participants were required to keep their eyes closed, maintain steady breathing, and refrain from swallowing or any bodily movements to minimize interference with EEG signals. The delivery tube of the taste stimulation device was precisely positioned 0.25–0.5 cm above the center of the subject’s tongue to ensure accurate delivery of stimulus solutions into the oral cavity. Each taste stimulation trial began with a randomly selected sweet solution, with participants unable to visualize the solutions. According to the experimental design, both sweet solutions and distilled water were randomly connected to the taste stimulation device to prevent potential bias.

Experimental preparation:

(1) The experimental environment should remain quiet to ensure that EEG detection data was not affected by ambient noise, and strong light sources should be avoided to prevent discomfort for experimenters. Before each experiment, the laboratory should be cleaned and ventilated to eliminate any odors.
(2) Participants should maintain good sleep quality and mental state on the day of the experiment, avoid consuming coffee, spicy foods, or neuroactive drugs, and refrain from using perfumes or other strong fragrances. We used an actigraphy to monitor total sleep time, sleep efficiency, sleep latency, and the number of awakenings. A subject is considered to have obtained sufficient sleep when the following criteria are collectively met: total sleep time reaches or exceeds 7 h, sleep efficiency is above 85%, sleep latency falls between 10 and 30 min, and nighttime awakenings are infrequent (typically fewer than 10 times throughout the night). Before the experiment, participants should be informed of the experimental procedure and key precautions.
(3) To prevent data distortion due to excessive scalp resistance, participants should ensure their hair is clean and avoid using excessive amounts of oily conditioners. They should also eat on schedule, with the experiment conducted one hour after a meal to prevent residual taste perception from affecting the data.
(4) The experimental environment should be strictly electromagnetically shielded, ensuring that no electronic devices other than the experimental equipment are present in the laboratory.

**Figure 2:**
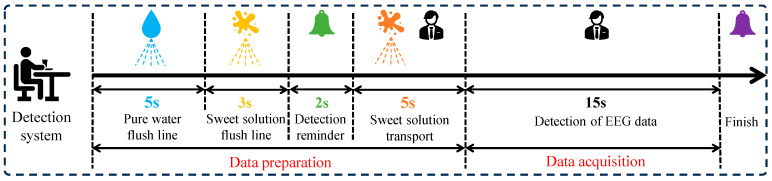
The data acquisition process in a parallel experiment.

**Figure 3:**
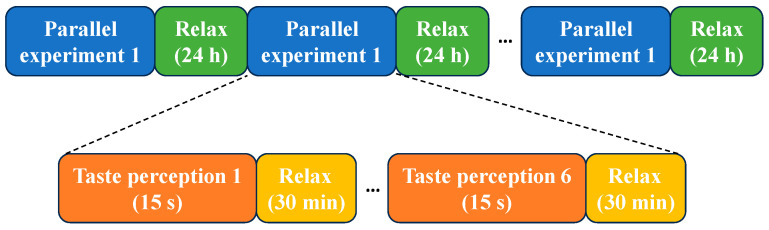
Experimental protocol for EEG-based sweet taste perception.

Experimental process:

(1) Distilled water rinse: Before each experiment began, pressing the switch will activate the distilled water rinsing mode of the taste stimulation device. Distilled water was rapidly delivered to the nozzle for 5 s to flush the pipeline.
(2) Solution rinse: After the distilled water rinse, the device automatically switched to the solution rinsing mode, quickly delivering the solution to the nozzle for 3 s to ensure that all distilled water was expelled and the pipeline was fully filled with the experimental solution. Upon completion, a buzzer emits a 0.5 s soft beep to notify the participant that the experiment was about to begin.
(3) Solution stimulation: Two seconds after the rinsing process ends, the taste stimulation device switched to solution stimulation mode, delivering 2.5 mL of sweet solution to the participant’s tongue over 5 s at a flow rate of 0.5 mL/s. The participant held the solution in their mouth for 15 s to fully induce the sweet taste EEG signal while avoiding swallowing.
(4) End of stimulation: Once the EEG data collection was complete, the buzzer sounded again to indicate the end of the experiment. The participant then thoroughly rinsed their mouth by gargling three times with 50 mL of clean water. To prevent taste fatigue, the participant rests for 30 min before proceeding to the next experiment.
(5) Taste EEG acquisition: During the taste EEG acquisition process, each participant underwent six parallel experiments for a given sweet taste concentration, with a 24 h interval between each parallel experiment. The complete taste EEG acquisition process was illustrated in Figure 2 and Figure 3.

This experiment collected taste EEG data from 20 participants under five different sweetness stimuli, with an additional blank control group as a separate category. Each taste stimulus generated six parallel 15 s EEG recordings, with each taste EEG sample having a duration of 1 s and a sampling rate of 256 Hz. A band-pass filter was then applied in the 0.5–45 Hz [29]. In total, 10,800 samples (20 × 6 × 6 × 15) were obtained, where 20 represents the number of participants, 6 denotes the number of categories and experimental repetitions, and 15 corresponds to the number of samples acquired per experiment. Each sample is structured as a 21 × 256 data matrix.

### 2.3. EEG Feature Computation Module

**Figure 4:**
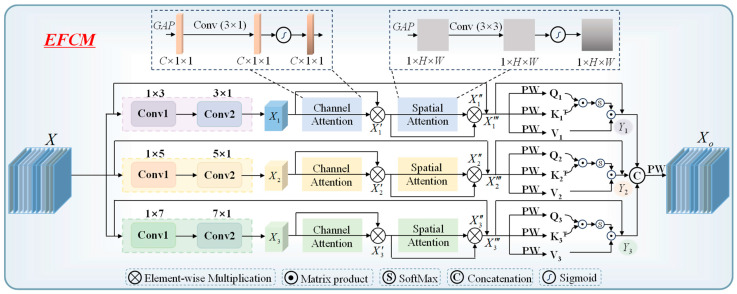
Illustrating the EFCM.

The data format of sweet taste EEG signals is 21 × 256, where 21 represents the number of electrodes, and 256 corresponds to the number of time sampling points. Since the electrical signals generated by different functional areas of the brain exhibit distinct characteristics, relying solely on a subset of electrodes may only capture EEG activity from specific regions, potentially missing important information from other brain areas. By integrating data from all electrodes, a more comprehensive analysis of overall brain activity can be achieved, providing a richer feature foundation for subsequent recognition tasks. EEG data inherently possess both temporal and spatial characteristics. Temporally, it is essential to consider the dynamic changes in brain signals over time. Spatially, attention must be given to the distribution and interactions between different electrodes. Based on these EEG characteristics, we propose the EFCM to enhance feature extraction and representation. The structure of EFCM is illustrated in Figure 4.

(1) Local feature computation

Convolutional computation is characterized by local connectivity and weight sharing, enabling dynamic feature extraction of localized EEG information through convolutional kernels. Given that EEG data have both temporal and spatial dimensions, we employ strip-shaped convolutions separately for deep local feature computation along each dimension. During computation, small convolution kernel sizes may not effectively capture correlations across multiple electrodes or long-duration sampling points, while large convolution kernels may fail to adequately compute local features between electrodes and across time sampling points. To address this, we design multiple convolutional branches, each utilizing different kernel sizes to fully extract local features from EEG data. Let the deep EEG data can be represented in $X\in {ℜ}^{C\times H\times W}$, the multi-branch convolutional computation can then be expressed as:

(1) $$X_{i}=Conv(Conv(X,1\times k),k\times 1)\;i=1,2,3\;k=3,5,7$$

where mathematical definition of *Conv* is:

(2) $$W(i,j)=\sum_{m=0}^{P-1}\sum_{n=0}^{Q-1}X(m,n)\times Y(i-m,j-n)\;\left\{\begin{array}{c} 0\leq i<P+M-1\\ 0\leq j<Q+N-1 \end{array}\right.$$

where the dimension of input matrix *X* is (*P*, *Q*), kernel matrix *Y* is (*M*, *N*). In deep local feature extraction, both informative and redundant features coexist. To address this, we employ adaptive computation to selectively enhance key deep EEG features across the channel and spatial dimensions. Channel Attention Mechanism: First, we apply global average pooling (GAP) to aggregate spatial information and generate channel-wise statistics (${ℜ}^{C\times 1\times 1}$). Next, a 1 D convolution (kernel size: 3 × 1) dynamically models inter-channel dependencies while maintaining dimensionality through appropriate padding and stride settings. The resulting features are then normalized via a Sigmoid activation function to produce channel attention weights. Spatial Attention Integration: The computed channel attention weights are multiplied with the original features to emphasize discriminative channels. This weighted output is subsequently processed to derive the final spatial attention result. The overall computation process can be summarized as follows:

(3) $$X_{i}^{\prime }=Sigmoid(Conv(GAP(X_{i}),3))⊗X_{i}$$

where the calculation formula of the Sigmoid activation function is:

(4) $$f(x)=\frac{1}{1+e^{x}}$$

Following the channel attention computation, we further enhance feature discriminability through spatial attention. First, GAP is applied along the channel dimension to generate spatial-wise statistics (${ℜ}^{1\times H\times W}$). To model adaptive relationships between different spatial positions, a 3 × 3 convolution is employed, with padding and stride configurations preserving the original feature dimensions. The resulting features are then normalized via a Sigmoid activation function to produce spatial attention weights. Finally, these weights are multiplied with the input deep EEG features to obtain the refined spatial attention output. The computational procedure is as follows:

(5) $$X_{i}^{\prime \prime }=Sigmoid(Conv(GAP(X_{i}^{\prime }),3\times 3))⊗X^{\prime }$$

Furthermore, we introduce residual connections to mitigate feature degradation during the local feature computation process [32]. The computation process can be expressed as:

(6) $$X_{i}^{\prime \prime \prime }=X⊕X_{i}^{\prime \prime }\;i=1,2,3$$

(2) Global feature computation

The self-attention mechanism in Transformer enables deep global feature computation [33]. It maps the input data linearly to generate three vectors: *Q* (Query), *K* (Key), and *V* (Value), which represent the deep representations of the input data. By multiplying *Q* with the transpose of *K* (*K^T^*), the interaction relationships between EEG features can be obtained. Then, SoftMax is used to quantify the importance of these features. Finally, the weighted features are multiplied with *V* to produce the self-attention computation results. The specific computation formula can be expressed as:

(7) Zi=SoftMax(QiKiTdK)⋅Vi i=1,2,3

where *d_k_* represents the dimensionality of the *K* vector, while *QK^T^* denotes the inner product computation between deep EEG features. Scale normalization is applied to reduce the magnitude of the data. In this work, we have already obtained local feature computation results through convolution, which serve as the feature mapping of deep EEG data. Therefore, we employ lightweight point-wise convolution (PW) to integrate deep local features, replacing the traditional large-parameter linear mapping method to generate *Q*, *K* and *V*. Where PW convolution is a computational form with a convolution kernel size of 1 × 1. Meanwhile, convert their dimensions to $Q\in {ℜ}^{C\times HW}$, $K\in {ℜ}^{C\times HW}$, and $V\in {ℜ}^{C\times HW}$, thereby enabling global dynamic features computation based on Formula (7). The computation process can be expressed as:

(8) $$Q_{i}=PWConv(X_{i}^{\prime \prime \prime })\;i=1,2,3$$

(9) $$K_{i}=PWConv(X_{i}^{\prime \prime \prime })\;i=1,2,3$$

(10) $$V_{i}=PWConv(X_{i}^{\prime \prime \prime })\;i=1,2,3$$

In the PW computation process, parameters are not shared, meaning that the PW parameters used to generate *Q*, *K*, and *V*. After obtaining the *Z* of each branch, we convert it to $Z\in {ℜ}^{C\times H\times W}$. Additionally, the number of self-attention computations in each branch remains consistent with the number of convolutional channels. Furthermore, we introduce residual connections to mitigate feature degradation during the global feature computation process. The computation process can be expressed as:

(11) $$Y_{i}=X_{i}^{\prime \prime \prime }⊕Z_{i}\;i=1,2,3$$

(3) Feature fusion

After completing the local and global features computations for each branch, the results need to be concatenated along the channel dimension. A lightweight PW computation is then applied to fuse the deep features after concatenation while ensuring that the output dimensions of the EFCM module remain consistent with its input. The specific computation process can be expressed as:

(12) $$X_{o}=PWConv(Concat(Y_{i}))$$

### 2.4. EEG Classification Networks and Hyperparameters

**Figure 5:**
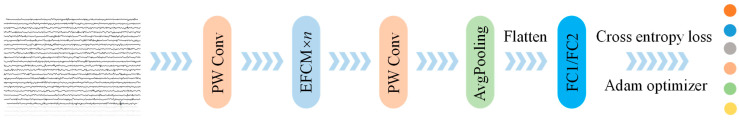
Network architecture of EFCC-Net.

Considering the characteristics of taste EEG data, we propose the EFCM. Based on EFCM, we design an EFCC-Net, with its structure illustrated in Figure 5. The specific design approach is as follows:

(1) PW convolution is applied to perform deep expansion of the raw EEG data, facilitating deep feature computation within EFCM. The number of channels in this convolution computation has been discussed in Section 3.2.
(2) EFCM is used to extract deep EEG features, where convolution operations capture local dynamic details, self-attention mechanisms focus on global dynamic relationships, and multi-branch computations enhance the richness of extracted features. This process ensures that the input and output dimensions remain unchanged. The number of EFCM units has also been discussed in Section 3.2.
(3) PW convolution is applied for dimensionality reduction of deep EEG features, enabling their fusion.
(4) Pooling operations with a kernel size of 1 × 2 are applied along the temporal sequence direction to reduce the complexity of feature mapping.
(5) Two fully connected layers are employed for adaptive recognition of taste EEG features at different sweetness concentrations. The first fully connected layer consists of 128 neurons, while the second layer contains 6 neurons.

The taste EEG stimulated by five different concentrations of sweet solutions and blank samples contained a total of 10,800 samples. For each sweetness concentration level, we divide the dataset into training and testing sets at an 8:2 ratio. During the training of EFCC-Net, parameter updates are performed using the cross-entropy loss function combined with the Adam optimizer set at a learning rate of 0.01. The training process consisted of 500 iterations with a batch size of 200. To analyze the stability of the EFCC-Net training process and assess the risk of overfitting, we employ 10-fold cross-validation to build the classification network. The mean and standard deviation of the results from the 10-fold cross-validation are used to describe the training outcomes of the sweet EEG signals. During testing, the classification network corresponding to the highest accuracy in the 10-fold cross-validation is selected as the optimal model to evaluate the generalization capability on the test set. Accuracy, precision and recall are applied to describe the classification results. Accuracy reflects the overall classification performance, while precision and recall can describe the classification situation within a class. The three-evaluation metrics are calculated as follows:

(13) $$Accuracy=\frac{TP+TN}{TP+TN+FN+FP}$$

(14) $$Precision=\frac{TP}{TP+FP}$$

(15) $$Recall=\frac{TP}{TP+FN}$$

where TP is true positive, TN is true negative, FN is false negative, and FP is false positive.

## 3. Results and Discussion

### 3.1. EEG Analysis of Sweet Taste Perception

**Figure 6:**
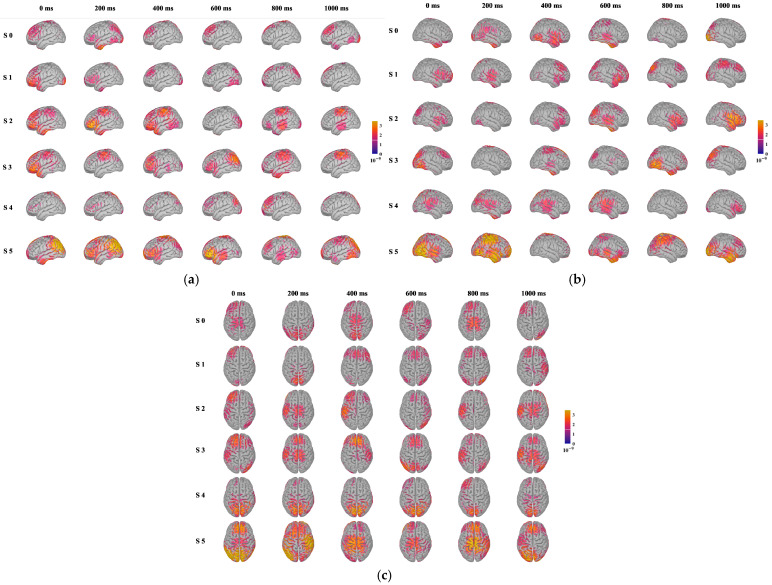
Results of brain region traceability under sweet stimulation. (**a**) Left, (**b**) right, (**c**) top.

Figure 6 displays the brain source localization results of participants under sweet taste stimuli of varying concentrations. Under six types of stimuli (S 0: pure water; S 1: 0.3 g sucrose/100 g pure water; S 2: 0.6 g sucrose/100 g pure water; S 3: 1.2 g sucrose/100 g pure water; S 4: 2.4 g sucrose/100 g pure water; S 5: 4.8 g sucrose/100 g pure water), the left, right, and top views of brain source localization at 0 ms, 200 ms, 400 ms, 600 ms, 800 ms, and 1000 ms are displayed for each stimulus. The neural activity in the brain exhibits significant systematic changes in intensity, spatial extent, and temporal dynamics. These patterns not only validate the feasibility of using EEG to distinguish sweet taste concentrations but also provide intuitive evidence for understanding the hierarchical neural mechanisms of sweet taste perception.

(1) Activation intensity and spatial distribution. As sweetness concentration increases, the intensity of brain activation strengthens significantly, and the activated regions expand from localized areas (such as the primary taste cortex) to broader brain regions (including higher-order association cortices like the prefrontal cortex). This “intensity-spatial” diffusion activation pattern closely aligns with known gustatory neural pathways. The primary taste cortex, mainly located in the insula and frontal operculum, is responsible for the initial encoding of basic taste qualities (including sweetness) and intensity [34,35]. The localized activation observed in the early stages of stimulation likely originates from these regions. As processing deepens, particularly under high-concentration stimuli, activation extends significantly to the prefrontal cortex, especially the orbitofrontal cortex, which associates with reward evaluation, decision-making, and affective processing [36]. This indicates that high-concentration sweet taste is not only perceived as an intense sensory signal but is also interpreted by the brain as a stimulus with high reward value, thereby engaging broader neural networks for affective and cognitive evaluation.

(2) Physiological interpretation of temporal dynamics. The temporal dynamics of brain activation reveal the hierarchical processing of sweet taste information in the brain: Early Stage (0–200 ms): High-concentration sweet taste stimuli elicit faster and stronger early responses. This stage primarily corresponds to the rapid transmission and initial encoding of taste information from glossopharyngeal receptors via brainstem nuclei (such as the solitary tract nucleus) to the primary taste cortex [37]. Stronger stimuli lead to more synchronized and larger-scale neuronal ensemble firing, manifesting as enhanced early activation. Mid Stage (200–600 ms): During this stage, the scope of activation expands, and coordination between different brain regions strengthens. This reflects the flow of information from primary sensory areas to higher-order association cortices, involving fine-grained processing of taste perception, comparisons with memory and prior experience, and the initial formation of affective valence (pleasant/unpleasant) [38]. High sweetness levels continue to evoke stronger responses at this stage, indicating its salience as a sensory signal that resonates broadly across neural networks. Late Stage (600–1000 ms): Under high-concentration stimuli, brain activity remains elevated during the late stage. This sustained neural activity may closely link to higher cognitive processes such as working memory (maintaining taste information online), integration of satiety signals, or ongoing evaluation of reward value [39]. In contrast, activation from low-concentration stimuli plateaus rapidly, suggesting limited depth and duration of cognitive processing.

Variations in sweetness concentration regulate EEG intensity, spatial distribution, and temporal dynamics, reflecting the brain’s hierarchical processing of taste stimuli. These findings suggest that EEG data can be effectively used for the qualitative identification of different sweetness levels.

The modulation of neural activation patterns by various physiological and psychological factors warrants further in-depth investigation. Physiological factors: Individual taste sensitivity serves as a key variable. For instance, the ability to taste phenylthiocarbamide or propylthiouracil correlates with polymorphisms in taste receptor genes. “Supertasters” may exhibit enhanced perception of flavors such as sweetness, accompanied by potentially intensified brain activation patterns. Additionally, metabolic states (e.g., hunger, which amplifies neural responses to the reward value of high-sugar foods) and endogenous hormone levels may modulate taste processing pathways. Psychological and experiential factors: Personal dietary preferences, cultural backgrounds, and past learning experiences shape “taste preferences.” Individuals with a particular fondness for sweet foods may show enhanced responses in reward circuits (e.g., nucleus accumbens, orbitofrontal cortex) to sweet stimuli, modulated by long-term experiences. Top-down psychological factors such as expectations, contextual settings, and brand perception can also significantly influence the neural representation of taste perception.

### 3.2. Optimization of EFCC-Net Structure

**Figure 7:**
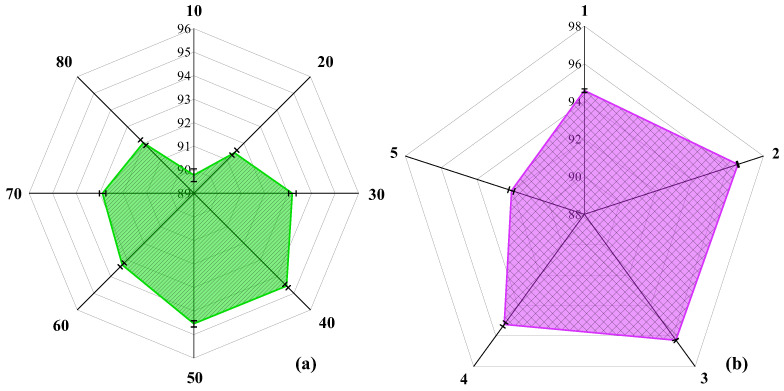
Optimization results of EFCC-Net: (**a**) Number of network channels, (**b**) Number of EFCMs.

To achieve optimal EFCC-Net performance for taste EEG classification across different sweetness concentrations, we optimize its structure. Section 2.4 shows the design principles and framework of EFCC-Net. During classification, the number of deep EEG channels and the number of EFCM units directly impact the network structure and, consequently, classification performance. For practical engineering deployment, where intelligent algorithms need to integrate efficiently with EEG detection, the designed network should be lightweight and structurally simple. Therefore, we evaluate deep channel numbers of 10, 20, 30, 40, 50, 60, 70, and 80 and also consider cases where the number of EFCM units is 1, 2, 3, 4, and 5. Figure 7a illustrates how different channel numbers affect EFCC-Net’s classification performance when only one EFCM is included. The results indicate that 40 channels yield the best classification accuracy. Too few channels fail to sufficiently extract deep features, while too many channels increase computational complexity without significant performance gains. Figure 7b shows the impact of varying EFCM numbers on classification performance. The results demonstrate that EFCC-Net achieves the highest classification accuracy with two EFCM units, suggesting that increasing network complexity does not necessarily enhance feature extraction or classification ability. Excessively complex network structures significantly increase computational costs and may even degrade classification performance. Through these structural optimizations, EFCC-Net with 40 channels and 2 EFCM units is identified as the optimal network architecture for taste EEG classification across different sweetness concentrations.

### 3.3. Ablation Analysis

During the EFCC-Net classification process, EFCM is a crucial module for obtaining deep EEG features and plays a significant role in classification performance. The EFCM design incorporates the following key components: (1) Multi-directional convolutional kernel operations: A 1 × K kernel extracts local dynamic features from the time series, while a K × 1 kernel models inter-electrode interactions. (2) Multi-branch computational pathways: The convolutional kernel size defines the local receptive field for deep EEG processing, thereby influencing the extraction of global dynamic features. (3) Integrated convolutional, self-attention, and residual mechanisms: The convolutional operations specialize in local dynamic feature encoding, self-attention captures global dynamic dependencies, and residual connections strengthen representational capacity. To fully validate the effectiveness of EFCM, we conduct three ablation experiments.

Table 1 presents the impact of convolutional kernel directions on EFCC-Net’s classification performance. When EFCC-Net includes only 1 × K or K × 1 convolution computations, it fails to jointly compute deep features across time series and electrodes, leading to insufficient feature extraction and a decline in classification performance. However, when EFCC-Net incorporates convolutions in both directions, it achieves optimal classification results, demonstrating the effectiveness of synergistic computation across temporal and spatial dimensions.

Furthermore, Table 2 shows the impact of different computational branches on EFCC-Net’s classification performance. The configurations are categorized into seven different computation scenarios. When using only one computation branch, EFCC-Net achieves higher classification performance at K = 5 and K = 7 compared to K = 1. This indicates that for high-sampling-rate EEG data, larger convolution kernels facilitate better local dynamic feature extraction. When two computational branches are included, larger convolution kernels further improve EFCC-Net’s classification performance, reinforcing the idea that larger receptive fields enhance local dynamic feature capture. Finally, when three computational branches are utilized, EFCC-Net achieves the best classification performance, confirming the effectiveness and stability of multi-branch computations.

Lastly, we examine the synergistic computation process of convolution, self-attention, and residual connections. Table 3 illustrates the impact of different computation modules on EFCC-Net’s classification performance. Since residual connections must be combined with convolution and self-attention computations, this discussion is divided into six different cases. Compared to using only convolution or self-attention, integrating residual connections results in a slight improvement in classification performance and enhanced model stability. Convolution captures local detail features, while self-attention computes global dynamic features. Ultimately, when all three computational processes are combined, EFCC-Net achieves the highest classification performance and stability, with an accuracy of 96.57%, a precision of 96.58%, and a recall of 96.53%, thereby proving the effectiveness of the designed EFCM module.

### 3.4. Performance Comparison of Classification Networks

**Figure 8:**
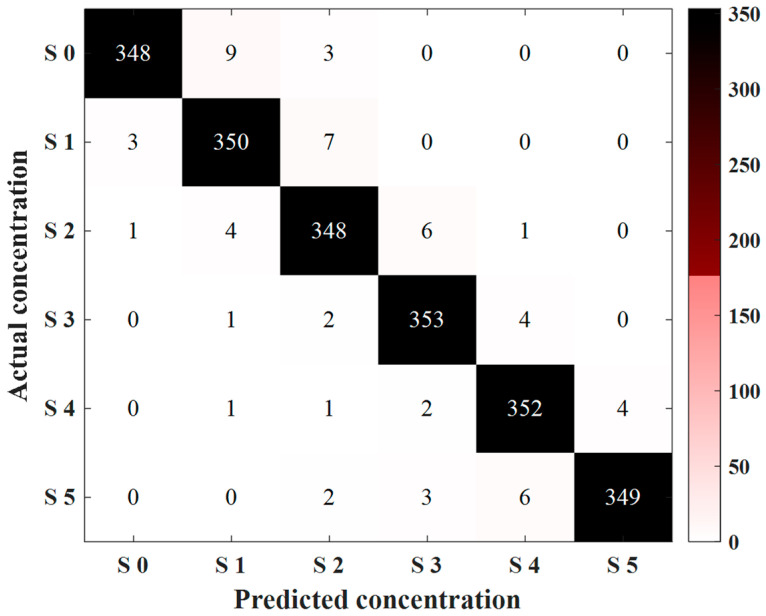
Classification result of the confusion matrix of the test set.

After demonstrating the rationality and effectiveness of EFCC-Net’s design, we compare it with other state-of-the-art classification methods, including lightweight convolutional neural networks, self-attention mechanism-based networks, and the most advanced EEG classification methods currently available. Table 4 shows the classification results. Among them, LeNet-5, MobileNet V2, and ShuffleNet V2 utilize lightweight convolutional computations to extract local features from sweetness EEG data and perform classification. However, since taste EEG data exhibits clear temporal and dynamic characteristics, failing to account for global temporal sequence dynamics results in the loss of critical features, thereby reducing classification performance. As a result, these methods do not achieve satisfactory classification performance. In contrast, Transformer and LeViT show significant improvements in classification performance, highlighting the superiority of global dynamic feature extraction over convolution-based local detail feature computation, leading to notable enhancements in classification results. Furthermore, when compared to the state-of-the-art EEG processing methods (as detailed in the introduction), EFCC-Net achieves the best classification results. Through ablation experiments, its rationality and effectiveness are validated, and when compared with other classification methods, its superiority is firmly established. Furthermore, based on the model corresponding to the best classification performance from the 10-fold cross-validation of EFCC-Net, tests are conducted on the test set. Figure 8 shows the confusion matrix of the test set, with an overall classification accuracy of 97.22%. EFCC-Net demonstrates excellent generalization performance, with only a small number of samples misclassified for each concentration level. Most of the misclassified samples are distributed among adjacent sweetness concentrations.

### 3.5. Future Research Directions

The focus of this study is to propose EFCC-Net for identifying taste EEG signals stimulated by different concentrations of sweet solutions. This approach utilizes a semi-objective method to achieve adaptive recognition of subjects under varying sweet taste stimuli. Without requiring subjects to provide sensory evaluation results, we can obtain identification outcomes based on EEG data corresponding to actual concentrations. In subsequent research, by incorporating actual sensory evaluation results, we aim to use artificial intelligence algorithms to achieve adaptive prediction of sensory sweetness scores, which constitutes our future research plan. Meanwhile, the identification of EEG data from different sweet taste concentrations serves as a foundation for further applications in the food industry, intelligent sensory evaluation, and multifaceted analyses such as preference, emotion, and memory. We provide a detailed explanation from the following aspects.

(1) Research for the food industry. EEG detection technology can serve as an objective tool for product development and optimization. For example, against the backdrop of global health trends advocating “sugar reduction,” this method can precisely evaluate the effectiveness of different sweeteners (e.g., natural sugar substitutes, artificial sweeteners) in mimicking the neural experience of sucrose sweetness. This aids in developing healthier products without compromising sensory quality.

(2) Training a sensory panel for the objective analysis of sensory test data. The identified neural features, such as specific spatiotemporal activation patterns associated with higher sweetness, can serve as neurophysiological benchmarks for screening and training panelists to achieve consistency in perceptual evaluations. Our EEG results can be directly compared with traditional sensory test outcomes. For instance, the intensity of brain activation in specific regions, including reward related areas, may correlate with quantitative ratings of sweetness intensity by panelists. Such comparisons will help bridge the gap between subjective verbal reports and objective neural correlates, validating and enriching the interpretation of both datasets.

(3) Investigate the interrelationships among factors such as preference, emotion, and memory to elucidate their underlying mechanisms. The obtained results can also be extrapolated to dimensions beyond basic taste recognition. Activations observed in brain regions such as the prefrontal cortex and insula, which are involved not only in taste perception but also in emotional processing, reward evaluation, and memory retrieval, suggest that our method may implicitly capture dimensions of preference, hedonic valence, and associated memories. Stronger or more widespread activation to specific concentrations may indicate not only sweeter but also potentially more pleasant or more familiar. Future research will explicitly combine our EEG classification method with self-reported preferences and emotional responses to fully leverage this potential for holistic product characterization.

### 3.6. Potential Limitations

A potential limitation and challenge lie in the relatively high sensitivity of EEG signals to environmental and physiological noise, as well as the fact that the current feasibility study is conducted in a limited controlled laboratory environment. To transform this method into a robust, large-scale consumer testing tool, these practical obstacles must be overcome, such as developing more user-friendly EEG systems and validating them across diverse populations. Furthermore, we will continue to explore EEG-based detection and analysis of other basic taste concentrations, advancing the application of EEG technology in food quality assessment.

## 4. Conclusions

Detecting and identifying consumers’ perception of sweetness helps guide the optimization of food formulations and product development. EEG analysis technology can capture changes in brain electrical activity in response to different sweet stimuli. By utilizing EEG signals to identify sweetness concentrations, it overcomes the limitations of traditional subjective evaluation methods, enabling an objective assessment of taste stimulus responses. In this work, a taste-EEG experiment with varying sweetness levels is designed to obtain semi-objective, physiological EEG data. Meanwhile, considering the data characteristics of deep EEG information, we propose an effective data processing method to achieve the identification of EEG signals corresponding to different sweetness levels. The main conclusions are as follows:

(1) With the increase in sucrose concentration from S0 to S5, there is a trend of enhanced activation intensity and expanded range in brain regions, which indicates the rule that the higher the sweetness, the stronger and more extensive the response in brain regions. In terms of the time dimension, low-concentration stimulation initiates brain region activation slowly and in a narrow range, while high-concentration stimulation can trigger broader activation more quickly. Under the stimulation of different taste concentrations, the subject shows obvious perceptual changes in the spatial and temporal brain regions, providing feasibility for taste perception and recognition based on EEG.

(2) Considering the complex temporal and spatial characteristics of EEG data, EFCM is proposed to compute deep EEG features. EFCM employs a multi-branch computational structure to extract features at different scales and utilizes convolutional and self-attention mechanisms within each branch to compute local and global dynamic features, respectively. Additionally, a residual computation structure is embedded to mitigate feature degradation during deep feature extraction. Ablation experiments confirmed the rationality and effectiveness of EFCM’s design.

(3) Based on the proposed EFCM, EFCC-Net is designed, consisting of PW convolutional layers, EFCM layers, pooling layers, and fully connected layers, exhibiting a notably lightweight architecture. Through structural optimization and comparison with state-of-the-art EEG classification methods, EFCC-Net demonstrate superior classification performance, achieving an accuracy of 96.57%, a precision of 96.58%, and a recall of 96.53%. By combining EEG experiments with EFCC-Net, EEG data corresponding to different sweetness levels are effectively identified.

In conclusion, EEG detection technology combined with EFCC-Net provides an effective technical approach for detecting and assessing consumers’ sweetness perception. Physiological EEG-based analysis offers a viable technical strategy for guiding food formulation optimization and product development.

## Figures and Tables

**Table 1:** Effect of different convolution computation directions in EFCM on the performance of EFCC-Net.

Case		Accuracy (%)	Precision (%)	Recall (%)
Conv(1 × K)	Conv(K × 1)			
√		95.09 ± 0.12	95.00 ± 0.13	94.96 ± 0.12
	√	92.45 ± 0.15	92.89 ± 0.14	92.74 ± 0.16
√	√	96.57 ± 0.03	96.58 ± 0.03	96.53 ± 0.03

**Table 2:** Effect of different computing branches in EFCM on the performance of EFCC-Net.

Case			Accuracy (%)	Precision (%)	Recall (%)
Branch 1	Branch 2	Branch 3			
√			91.22 ± 0.13	91.17 ± 0.14	91.02 ± 0.13
	√		94.37 ± 0.16	94.44 ± 0.16	94.25 ± 0.16
		√	94.89 ± 0.11	94.91 ± 0.10	94.80 ± 0.11
√	√		94.98 ± 0.13	95.13 ± 0.12	95.03 ± 0.13
√		√	95.22 ± 0.11	95.29 ± 0.11	95.31 ± 0.11
	√	√	95.49 ± 0.11	95.55 ± 0.12	95.57 ± 0.18
√	√	√	96.57 ± 0.03	96.58 ± 0.03	96.53 ± 0.03

**Table 3:** Results of the impact of different computing modules in each branch of EFCM on the performance of EFCC-Net.

Case			Accuracy (%)	Precision (%)	Recall (%)
Convolution	Self-Attention	Residual			
√			89.26 ± 0.21	89.47 ± 0.20	89.55 ± 0.20
	√		92.39 ± 0.14	92.83 ± 0.13	92.67 ± 0.13
√		√	89.85 ± 0.18	89.74 ± 0.19	89.83 ± 0.18
	√	√	93.12 ± 0.11	93.01 ± 0.11	93.02 ± 0.12
√	√		94.54 ± 0.12	94.55 ± 0.14	94.66 ± 0.15
√	√	√	96.57 ± 0.03	96.58 ± 0.03	96.53 ± 0.03

**Table 4:** Performance comparison results of multiple classification methods.

Methods	Accuracy (%)	Precision (%)	Recall (%)
LeNet 5	88.75 ± 0.21	88.87 ± 0.22	88.43 ± 0.21
MobileNet V2	90.51 ± 0.21	90.41 ± 0.21	90.16 ± 0.21
ShuffleNet V2	92.07 ± 0.15	91.93 ± 0.16	92.08 ± 0.16
Transformer	94.04 ± 0.19	94.39 ± 0.19	94.20 ± 0.20
LeViT	94.18 ± 0.13	94.26 ± 0.11	94.20 ± 0.12
Ref. [15]	92.45 ± 0.17	92.89 ± 0.15	92.74 ± 0.16
Ref. [22]	94.35 ± 0.15	94.35 ± 0.15	94.28 ± 0.15
Ref. [23]	93.12 ± 0.20	93.01 ± 0.20	93.02 ± 0.20
Ref. [24]	94.27 ± 0.19	94.59 ± 0.19	94.38 ± 0.19
Ref. [26]	94.20 ± 0.19	94.47 ± 0.19	94.28 ± 0.19
Ref. [27]	94.80 ± 0.11	94.74 ± 0.12	94.70 ± 0.13
EFCC-Net	96.57 ± 0.03	96.58 ± 0.03	96.53 ± 0.03

## Data Availability

The original contributions presented in this study are included in the article. Further inquiries can be directed to the corresponding authors.
